# Remarks on Mr. Lucas's Practical Observations on Amputation

**Published:** 1786

**Authors:** Lancelot Haire

**Affiliations:** Surgeon at Southminster, in Essex, Member of the Corporation of Surgeons of London, and formerly Assistant Surgeon to the Royal Hospital at Haslar


III. *Remarks on Mr. Lucas's practical Obſervations on Amputation. Communicated in a Letter to Dr.* Simmons, *F. R. S. by Mr.* Lancelot Haire, *Surgeon at Southminſter, in Eſſex, Member of the Corporation of Surgeons of London, and formerly Aſſiſtant Surgeon to the Royal Hoſpital at Haſlar.*

HAVING lately peruſed, in the London Medical Journal *, ſome practical obſervations on amputation by Mr. Lucas, an ingenious ſurgeon at Leeds, which, in ſeveral reſpects, differ from my experience and ideas on this ſubject, I take the liberty of communicating to you a few remarks on Mr. Lucas's paper, to be inſerted, if you think proper, in the ſame work.

* Page 225.

My aim is, by a liberal and difpaffionate inveftigation of Mr. Lucas's obfervations, to examine their claim to merit, and to rectify error, as far as my abilities will permit. This confideration alone has enticed me, from my prefent obfcure fituation, to appear, for the firft time, as a writer; and when I declare that I have not the pleafure of being perfonally known to Mr. Lucas, I hope my motives will not be confidered as hoftile, or as tending to depreciate his profeffional merit in the eyes of the world.

" In cafes," fays Mr. Lucas *, " where the " life of the patient is not immediately con- " cerned, every remedy which the fkill of the " furgeon can fuggeft fhould be preferred to " operation." But it is certain that cafes occur daily where the life of the patient is not immediately concerned, and in which it would be the height of imprudence to delay the operation till it is. We have feen many fuch patients whofe tibias were difeafed, from the havock made in the foft parts, by true fcorbutic ulcers, who, with proper nourifhment, might be fupported for months, and yet, in the end, nature be as far from accomplifhing her purpofe

* Page 225.

as as in the beginning. Nature, I ſay, becauſe I am afraid ſkill will avail little in effecting a cure where the whole anterior part of the tibia has been bare a very conſiderable time, and without the leaſt apparent exfoliation.

It has been propoſed, in diſeaſed bones of this kind, to apply the actual cautery, or the trepan, in order to promote a ſeparation. Of the two, perhaps, the burning the diſeaſed bone is to be preferred. It ſeems to act, by inducing ſuch an inflammation in the adjacent ſound parts as will enable the conſtitution to bring about an exfoliation more quickly: but till the operation of the trepan can be confined to the diſeaſed portion, without injuring the ſound, it ought to be rejected.

In ſuch caſes every remedy that can be ſuggeſted commonly fails, and nature, with all the aſſiſtance that can be afforded her, is, in general, inadequate to the cure. Surely, then, it would be better to determine at once on amputation, than to protract the ſufferings of patients three or four months unneceſſarily.

I muſt not be underſtood as being an advocate for precipitate amputation — quite the reverſe. The doctrine I wiſh to inculcate is, that when we have got our patient in a proper ſtate

 for for an operation, it is our duty to perform it without delay, notwithſtanding his life is not immediately concerned.

"In caſes of accident," continues Mr. Lucas, "it is frequently neceſſary to decide "within a few hours, or no ſafe opportunity "for the operation may afterwards offer *." This doctrine, taken in too wide an extent, and inculcated by Mr. Pott, has been the cauſe, I fear, of many precipitate amputations. I will advance a ſtep farther, and declare, that, in caſes of accident, I believe more people loſe their lives and limbs by too early operation, than otherwiſe would by delaying it, and patiently waiting the event. Had I not been in a ſituation, during the late war, of ſeeing many gun-ſhot wounds brought to the hoſpital at Haſlar, I would not have ſpoken thus confidently. When a patient dies from a local injury, with the limb on, we are apt to blame ourſelves for not having operated; but it does not follow that the operation would have ſaved him. I have often ſeen the reverſe; and, in moſt caſes of this kind, I believe immediate amputation would have been as fatal as delay.

* See page 225.

In the navy, during the war, it was the conſtant cuſtom for the ſurgeon and his mate to be ſtripped in the cockpit, ready to operate the moment the poor men were carried off deck. In the heat of action, perturbation of mind, and confuſion, natural upon ſuch occaſions, ſurely a more improper time could not have been choſen. What was the conſequence? The fatality was incredible. — I ſhould think myſelf happy could I prevail on our naval ſurgeons to delay operations of conſequence at leaſt ſome hours after an engagement, till they and their patients cool, and have leiſure to deliberate upon what they are about. I am well aware that caſes of hæmorrhage require immediate attention; but when proper means are uſed, they ſeldom render inſtant operation neceſſary.

But at the end of a month or ſix weeks, in caſes of accident, it often becomes neceſſary to amputate. People then commonly exclaim, It was a caſe for immediate amputation at firſt, and why was the patient kept all this time in ſuſpence and miſery? They ſeldom conſider that this is the very time (when the violent inflammation and ſymptomatic fever, the conſequence of the accident, are abated) to amputate tate a limb with moſt ſucceſs; for the effects of the operation will then be little felt by the conſtitution.

What I wiſh to infer particularly, from what has been ſaid, is, that there are many chronic caſes in which the life of the patient is not immediately concerned, and in which the operation may be improperly protracted.

But, in caſes of accident, the operation is often too haſtily determined on in the beginning, when it might be performed with a little ſuffering or riſk, and more ſatisfaction, after having waited the event of the firſt inflammation: for whoever has ſeen patients loſe their limbs in full health will acknowledge, that their danger is not ſlight, and the proſpect of ſaving by this practice even one limb in twenty will be a laſting pleaſure. The ſubject being of importance, and the reverſe of this practice being, I fear, too generally followed, has induced me to be thus prolix.

"Blood veſſels, in the vicinity of the af-"fected part, may be ſo numerous, and ſo "much enlarged, as to require the removal of "the limb at a greater diſtance than might "otherwiſe be choſen *."

* See page 126.

This

This is to be underſtood with very conſiderable limitations. It ſhould not induce us to operate above knee where we otherwiſe would below, nor above the elbow, when we have it in our power, except for this doctrine, to ſave the joint.

Admitting the veſſels to be more numerous as to their ſize, (though I think this is with difficulty to be determined previouſly to the operation) ſtill they will only require more care and attention in taking them up; and the ſatiſfaction of having preſerved the joint will more than counterbalance the additional trouble, conſidering the riſk is not a great deal more.

We are told by Mr. Lucas, that "the ſubſtitute propoſed by Mr. Parke, for amputation, in caſes of diſeaſed joints, does him the greateſt credit*." I cannot help, however, being of a very different opinion. The mode of treatment alluded to conſiſts in diſſecting out the diſeaſed part, after the bones have been ſawn through, both above and below the joint. I once ſaw this performed upon the dead ſubject, but never wiſh to ſee it again on the dead or living. Surely, after ſubmitting to

* Page 226.

ſuch an operation, the poor patient's proſpect of advantage ſhould not be viſionary. Granting that callus forms in all the intermediate ſpace between the ends of the bones, ſtill we are likely, after a very doubtful operation, to have a ſtiff, unweildy limb, more uſeleſs than a wooden one; and if the callus ſhould not form the whole length, it muſt be ſtill worſe As this operation, as a ſubſtitute for diſeaſed joints, has been ſtrenuouſly recommended, I have embraced the preſent opportunity of entering a proteſt againſt it.

About a twelvemonth ago I ſaw, by accident, a ſingular caſe of fracture. The account the man gave me was, that he had been a ſailor on board a merchant ſhip going to the Weſt Indies; that he had fallen down on the paſſage, and broke his arm a little above the elbow. They had no ſurgeon on board, he ſaid, and after lying upwards of a month, without any proper aid, he was ſent on ſhore for advice; but it was then too late. When I examined him, there was a complete ſeparation of the os humeri full two inches, forming an artificial joint at the place which had been fractured. The fore arm was ſomewhat waſted, totally uſeleſs, and hung in a dead, floating manner;

 the the two parts of the arm were kept together by nothing but the muſcles and integuments, whoſe connection ſomewhat reſembled that of a flail. The inferior portion of the bone was turned in and out of a line with the ſuperior end of the fracture. This would ſeem to have proceeded from unſkilfully raiſing, and ſupporting the arm at the elbow out from the body; and this poſition, by throwing the ends of the fractured bone quite out of a line, might have tended to prevent the union. It had been originally a ſimple fracture, and it felt ſmooth and even at that time.

Had not this man begged as a vagrant, I am perſuaded he had rather have been without the incumbrance of the preſerved limb.

The chief deſign of Mr. Lucas's obſervations, in the paper I am conſidering, ſeems to be, to revive the almoſt-forgotten flap operation. The limits of your very uſeful Journal will not permit me to enter into the merits of that operation, unleſs its advocates ſhould farther inſiſt on its ſuperiority over the eſtabliſhed practice of operating by a circular inciſion.

I ſhall beg leave, however, to recommend a re-conſideration of ſeveral parts of his paper to the ingenious author, and I flatter myſelf, he he will think with me, that there are minutiæ in every profeſſion beneath the notice of a man of genius.

How far the introduction of line and rule, and, of courſe, of mathematical calculations, in ſurgical operations, is compatible, is ſubmitted, with deference, to the opinion of the public. When we are taught to believe that it is neceſſary to take the dimenſions of a limb, in order to determine the exact proportion of integuments to be ſaved, in an amputation, it tends to give us an erroneous and mean opinion of nature's reſources in caſes of exceſs or diminution of parts. Numbers of inſtances have fallen within my knowledge where the overplus of the integuments was from half an inch to an inch, yet, during the cure, they always retracted, and never prolonged it. A trifling deficiency may eaſily be made up by bandage. What happens daily in caſes of pregnancy, and dropſy, in the hare-lip and other operations, will ſufficiently illuſtrate the two points of deficiency or redundancy.

We are preſented with an inſtance of the bone being found, at the firſt dreſſing, protruded through the ſkin; and the concluſion the author draws from it is, that "an undue

" proportion

"proportion of the integuments had been pre-
"served *."

I have seen many such cases, and aver, that they uniformly proceeded, in my judgement, from an unguarded separation of the integuments from the bone, higher up than where we saw through. This is easily done, particularly on the fore part of the tibia, where the cellular membrane is attached so very loosely; and I am happy to have this opportunity of cautioning against committing it, as a profusion or exfoliation is very liable to happen from a bone being incautiously laid bare above where we saw through. I know of no undue proportion of integuments that could occasion this, except improper pressure should be made at the same time.

We are ordered † attentively to reserve muscular parts, in order to protect the end of the stump; but if pressure be afterwards made on its end, it will generally wear as thin as if no such reservation had taken place, owing to the pressure producing an absorption; and, for this reason, I think the limb more safely supported any where else than at its end.

Turning the edge of the knife upwards, in order to prevent the adipose membrane from starting out, is mentioned as an idea of Mr. Alanson's *. This gentleman has already introduced a very valuable improvement into surgery; but

"Est modus in rebus; sunt certi denique fines,

"Quos ultra citraque nequit consistere rectum †."

Our author prefers the tenaculum for laying hold of the vessels: perhaps the dissecting forceps would be no improper auxiliary in many cases.

The dimensions of a bolster ‡, in order to make a more effectual compression on the artery, is given; — a linen roller, done up tight, has always succeeded in the cases that have fallen under my observation.

I recollect an assertion of this gentleman's on another occasion §, that, in this country, amputation is *rarely* performed but on scrophulous patients. It may be so at Leeds; but I

* Page 230.

† Horat. Sat. lib. 1.

‡ Page 237.

§ Medical Obs. and Inq. Vol. V. page 325.

could oppoſe to this the practice of all the naval hoſpitals in the kingdom, in which I will venture to aſſert, that almoſt nineteen in twenty of the amputations are for true ſcorbutic ulcers, particularly in the one in which I had the honour of being employed during the war, where the average number of patients was upwards of a thouſand, and conſequently operations muſt have been numerous. I could appeal, as authority for this fact, to ſeveral gentlemen, my colleagues, now ſettled in different parts of the kingdom.

As England is already celebrated, by foreigners, for conſumptions, they might reaſonably infer, from this aſſertion, if well founded, that ſcrophula, in one ſhape or other, deſtroys half its inhabitants.

In having thus examined ſome of Mr. Lucas's obſervations with freedom, I hope I have not exceeded the bounds of decorum, as I diſclaim any intention of giving perſonal offence upon this occaſion.

With reſpect to the ſucceſs of healing the ſtump by the firſt intention, at Haſlar hoſpital, it always exceeded our expectations in emaciated ſubjects, and when the conſtitution had been previouſly much reduced; but where it was performed on subjects in full health, in cases of accident, it did not succeed ; a violent inflammation and swelling coming on, burst the adhesions, the muscles shot out, and occasioned almost as open a stump as in the old method of operating. The ligatures sometimes became troublesome, and retarded the cure. An intimate friend of mine, a surgeon of great abilities, proposed to cut the ends of them off close to the knot, and thus leave them to themselves.

By following this plan, we have seen stumps healed in the course of ten days. The short ligature, thus left in, commonly made its way out by a small opening, in a short time, without any trouble, or the patient being sensible of pain.

*Southminster, Essex,*
Nov. 1st, 1786.

IV. *An*

